# Infection in the intensive care unit alters physiological networks

**DOI:** 10.1186/1471-2105-10-S9-S4

**Published:** 2009-09-17

**Authors:** Adam D Grossman, Mitchell J Cohen, Geoffrey T Manley, Atul J Butte

**Affiliations:** 1 Department of Bioengineering, Stanford University, Stanford, 94305, California, USA; 2 Department of Surgery, University of California San Francisco, San Francisco, 94110, California, USA; 3 Department of Neurosurgery, University of California San Francisco, San Francisco, 94110, California, USA; 4 Center for Biomedical Informatics Research, Stanford University, Stanford, 94305, California, USA

## Abstract

**Background:**

Physicians use clinical and physiological data to treat patients every day, and it is essential for treating a patient appropriately. However, medical sources of clinical physiological data are only now starting to find use in bioinformatics research.

**Results:**

We collected 29 types of physiological and clinical data on a minute-by-minute basis from trauma patients in the intensive care unit along with whether they contracted an infection during their stay. Dividing the patients into two groups based on this criterion, we determined that the correlational network amongst pairs of physiological variables changes based on whether the patient contracted an infection.

**Conclusion:**

Examining the variable pairs with the largest change in correlation across groups reveals potential changes in the way our treatments affect the patient's physiology and in how our bodies react to physiological insults. These findings highlight the usefulness of physiological informatics and suggest new relationships to study while also validating previously reported relationships.

## Background

While physicians have been making use of physiologic data for decades, these measurements have not always been collected and stored electronically for research use. Largely due to the advent of electronic medical records and improved methods of extracting data from patient monitoring equipment and paper charts, we can now obtain significant amounts of clinical data both during and after treatment [1-4]. This points to a relatively new source of data that has been made available to both clinicians and informatics researchers in ways that were not possible previously.

One of the main thrusts of informatics research using clinical data has been to provide greater context for defining disease using physiological and treatment data [5,6]. Use of clinical data for basic biological research has been successful in linking physiological measurements to already known genetic markers of disease [7], and has yielded biomarkers related to human aging [8].

One area where there seems to be less research is in the physiological modelling of humans undergoing intensive care in a hospital setting and the various conditions these patients encounter. It is this gap that we seek to fill by providing data showing that even basic physiological models of patients in intensive care are changed under conditions of infection. We also show how treatments administered during intensive care can affect physiology in different ways depending on the state of the patient.

## Methods

### Data collection

Our data were obtained from 19 patients who were admitted to the trauma intensive care unit (ICU) at San Francisco General Hospital from May 2004 to June 2005. These patients were generally heavily injured, with average stays in the ICU of 21 days and an average hospital stay of 36 days total. Upon admission to the ICU, patients were connected to standard ICU monitors and ventilators as necessary, and were also monitored with an experimental muscle microdialysis catheter capable of measuring metabolic parameters such as glucose, lactate, and pyruvate.

Data were collected from all the above sources, plus blood gas analysis and laboratory measurements of various standard ICU biomarkers. The types of biomarkers collected are listed in Table 1. Data from the ICU monitors were collected at one-minute intervals and stored on a dedicated server. The remaining data were collected from the patient's medical records and annotated with the time of each measurement. The resulting data set contained up to 92,000 measurements of each of 29 types of physiological measurement, yielding approximately 2.7 million total data points. Patients' records were annotated as to whether they developed multiple organ dysfunction or an infection. Infectious complications included pneumonia, bacteremia, sepsis, abscess, urinary tract infection, wound infection, infected decubitus ulcer, infected hardware, meningitis, and osteomyelitis. Patients were tracked until they either died or were discharged.

**Table 1:** Types of data collected and their abbreviations

**Symbol**	**Definition**
PaO_2_/PCO_2_	Arterial O_2_/CO_2 _partial pressure
MAP	Mean arterial blood pressure
HR	Heart rate
pmO_2_(temp)	Muscle oxygen level (temperature)
spO_2_	Oxygen saturation percentage
FiO_2_	Fraction of inspired oxygen
GLUC.SER	Serum glucose
Ph	Blood PH
PF	PaO_2_/FiO_2 _ratio
BD	Base deficit
mLactate	Muscle lactate concentration
mGlucose	Muscle glucose concentration
mGlutamate	Muscle glutamate concentration
mPyruvate	Muscle pyruvate concentration
mLP	Muscle lactate/pyruvate ratio
Lactate	Serum lactate
glucose.value	Bedside glucose reading
compliance	Mechanical lung compliance
peep	Positive end expiratory pressure
minvol	Volume of air per minute
coretemp	Core temperature
CVP	Central venous pressure
Hb/HCT	Hemoglobin/hematocrit
Chloride	Serum chloride
BUN	Blood urea nitrogen
Cr	Serum creatinine

### Data analysis

Prior to data analysis, the muscle microdialysis data required correction to reflect its semi-continuous sample collection method. As the microdialysis catheter would continuously collect extracellular fluid from the muscle for approximately 30 minutes between readings, each reading was an average of the metabolites collected over that span. To fill in the missing data from this data source, we performed linear interpolation between successive readings of the dialysate composition.

Data were analyzed by calculating Pearson correlation coefficients for each pair of variables under different physiological conditions (406 pairs in total). We grouped each patient according to whether he or she contracted an infection during his or her stay in the hospital. In our cohort 11/19 patients (58%) contracted an infection. The data used to calculate the correlation for each variable pair were all those rows containing data for both variables of that pair, i.e. "pairwise complete observations". While this can yield a correlation matrix that is not positive definite, we are concerned with the coefficients themselves rather than manipulating the resulting matrix.

Correlations were considered significant if the p-value derived from Student's t-test was less than 0.05 after correcting for multiple comparisons using the Holm-Bonferroni method. Taking those correlations that were significant in both the infection and non-infection groups and intersecting them resulted in the candidate set of variable pairs to further investigate. We then take the absolute value of the difference between the candidate correlations in our set and only consider correlations after applying a cut off value for the difference of 0.4.

## Results

**Figure 1:**
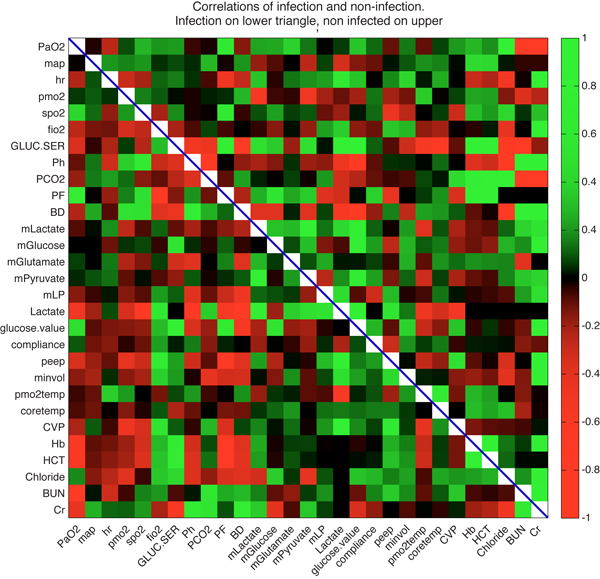
**Variable correlations**. Correlations between physiological variables for patients who did (lower triangle) and did not (upper triangle) contract an infection. Colour indicates the magnitude and direction of the correlation as shown in the colour bar at the right. Variable names are given on the axes.

The overall correlations among biomarker data are shown in Figure 1, with all correlation coefficients shown regardless of statistical significance. One can easily discern variables that switched their correlation direction by observing the colour change from red to green in the matrix transpose position. For example, muscle oxygen partial pressure and heart rate positively correlate (weakly) in the infection case (position 4 down, 3 right from top left) and anti-correlate (again, weakly) in the non-infection case (position 3 down, 4 right from top left). Some of the black locations indicate a lack of data while others indicate absence of correlation. In both cases, we do not consider them further here.

**Figure 2:**
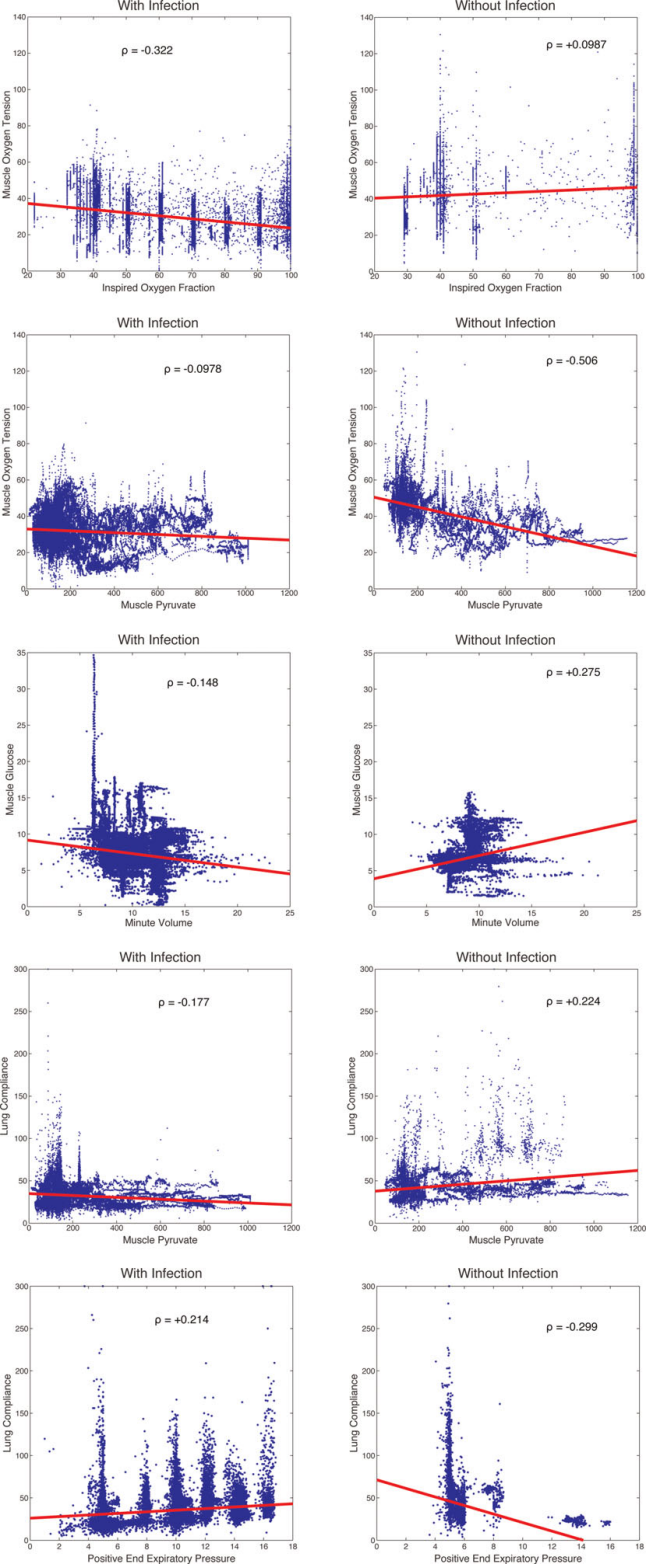
**Five largest changes in correlation**. Raw data and regression lines for each of the top five changed relationships in our study. The top row shows data from the non-infection group and the bottom row shows the infection group.

Given the large number of comparisons here (802 between the two cases, accounting for those comparisons lacking data), it is reasonable to ask what are the most extreme examples of changes in correlation between the two conditions. Taking the absolute value of the difference in correlation, the top five are shown in Table 2. The raw data and regression lines for each of the correlations are shown in Figure 2. In total, regardless of statistical significance, 164 out of 401 correlations changed direction between conditions.

**Table 2:** Correlation coefficients of the variables with the largest change in correlation between conditions

**Variable Pair**	**ρ with infection**	**ρ without infection**
FiO2/PmO2	-0.332	0.0987
mPyruvate/PmO2	-0.0978	-0.506
minvol/mGlucose	-0.148	0.275
compliance/mPyruvate	-0.177	0.224
peep/compliance	0.214	-0.299

We then consider the correlation pairs for which each correlation was significant. Four of the top five changes in correlation involved a switch in the direction of correlation in the two conditions – i.e. they are positively correlated in one condition and negatively correlated in the other.

## Discussion

Upon splitting the physiological data into patients who did and did not contract infections during their hospital stay, we have revealed that several physiological variables change the direction of their correlations. Here, we will explain these changes via physiology and general standards and methods of treatment in the ICU. It should also be noted that we are trying to explain relationships that do not indicate a direction of causality. As a tool, however, we will hypothesize causality in some of the following discussion.

### Fraction of inspired O_2 _vs. muscle O_2 _content

In the non-infection case, as the fraction of oxygen inspired increases so too does the resulting partial pressure of oxygen throughout the remainder of the body's tissues, as expected. We would further expect the partial pressure to saturate very quickly with increasing oxygen inspiration, as in healthy individuals oxygen saturation is above 95% [9].

In the case of infection, though, there are many potential physiological changes, ranging from hypotension to impaired oxygen transfer, and other problems that could also lead to hypoperfusion. Sair et al. showed that in a rat model of endotoxemia the effect of increasing FiO_2 _on muscle oxygen saturation is significantly reduced in the infection group [10]. Because there were no observable differences in tissue perfusion between the two groups in that experiment, it is possible that our data showing a reversal in the correlation are due to reduced tissue perfusion combined with the reduced oxygen delivery capability despite normal perfusion as shown by Sair.

Ikossi et al. showed that lower PmO_2 _was strongly associated with worse outcomes in the ICU, including infection and multiple organ failure [11]. In patients who appeared to be well resuscitated by normal measures (MAP, heart rate, BD, and PaO_2_) reduced muscle oxygen content predicted worse outcomes and increased complications. Because this study used oxygen sensors placed in the deltoid muscle, this indicates a likely decrease in perfusion to distal organs using muscle as a proxy. This is despite the clinicians attempting to increase perfusion by increasing FiO_2_.

It is also relevant that inspired oxygen fraction is controlled by the ICU staff. Fraction of inspired O_2 _is primarily increased to compensate for decreases in tissue oxygen saturation. So the prior expectation is that patients in the ICU, especially those with impaired lung function (as those with pneumonia often have), will have their oxygen intake increased as their tissue oxygen pressure decreases, leading to the negative correlation seen in our data. The most likely explanation for the change seen in the two conditions is that the infection reduces the coupling of inspired oxygen fraction to oxygen present in tissue, and then clinical intervention results in increasing FiO_2 _in the face of decreasing PmO_2_.

### Muscle pyruvate vs. muscle O_2 _content

In both the infection and non-infection groups, muscle pyruvate and muscle oxygen pressure are anti-correlated. In the non-infection group, the correlation is stronger, indicating a tighter connection between oxygen delivered to the tissue and the removal of pyruvate form the tissue via aerobic respiration. This could indicate consequences of infection similar to those proposed in the previous section – i.e. patients with infections have reduced ability to deliver oxygen to the tissue and so the rate of metabolism is less tightly coupled to the delivery of oxygen than in trauma patients without infection. This may point to mitochondrial dysfunction as a culprit in this relationship.

### Minute volume vs. muscle glucose

It has been reported that glucose metabolism is altered in patients with sepsis, and furthermore that in critically ill patients reduced glucose can be seen largely as a marker for disease severity [12]. We can therefore postulate the following. Patients with infections are clearly more ill than those without infections, suggesting that they will have reduced levels of glucose in their tissue. That these patients are sicker may also indicate a need for increased ventilation, either to expedite removal of CO_2 _from the blood or increase the ability of oxygen to enter the bloodstream. So in patients with infections, we would therefore expect to see the minute volume to be negatively correlated with tissue glucose.

This result may also not be explainable by traditional physiology. Glucose is tightly controlled by ICU physicians, so these results are also clouded by that intervention. We have shown here a relationship that does not yet exist in the literature, so this defines a new physiological relationship between these two variables. The difference in the relationship between these variables, then, may be an indicator of severity of sickness or a predictor of outcome rather than something to be explained.

### Lung compliance vs. muscle pyruvate and PEEP

Lung compliance is a variable measured by mechanical ventilators. It is the ratio of the change in lung volume in response to a change in air pressure. Several factors affect lung compliance, which include the amount of surfactant present in the alveoli and pathologies, such as fibrosis.

In patients with infection, PEEP and compliance co-vary, while the opposite is true for non-infection patients. It has been shown that in rats given a dose of lipopolysaccharide (an antigen produced by the that bacteria cause sepsis) who also received increased constant airway pressure had increased lung damage [13]. Experience dictates that sicker patients (like those with pneumonia) have stiffer and less functional lungs, which would necessitate the use of a higher PEEP to recruit otherwise collapsed alveoli. This increases the apparent lung volume for a given pressure in the sicker patients resulting in a measured increase in lung compliance. It is unclear why this should be the reverse in less sick patients, but in looking at the scatter plot it is clear that the majority of the data have a PEEP of approximately 5, with fewer samples at higher values.

Again, there is no simple reason why lung compliance should be at all related to the concentration of pyruvate in muscle tissue. It is not a problem that this relationship is not readily explainable, since this is now a new physiological relationship that we've defined. To be complete, however, a hypothesis is as follows. Lung compliance measures, in part, the ability of the alveoli to open and accept new air in response to increasing inspiratory pressure. In patients with infection, as compliance increases, muscle pyruvate decreases. This results in a greater volume of air being available to supply oxygen, thereby increasing the potential delivery rate of oxygen to the blood and peripheral tissues. Increased oxygen allows aerobic respiration to proceed faster, decreasing the amount of pyruvate present in the system.

### Application as a decision tool

It should be noted that these results could find potential use as the basis for a decision tool to warn clinicians of impending infection. While our data set did not allow us to test this application, future work could include testing our results on novel data. This highlights the overall utility of pursuing physiological informatics and modelling using clinical data.

## Conclusion

We have shown that physiological networks can be constructed from clinical measurements from an intensive care unit, and the topology of these networks can change as a patient contracts an infection. Between these two conditions many variables changed the strength or direction of their correlation. The five pairs of variables with the largest magnitude of change were:

• FiO_2 _vs. muscle oxygen content

• Muscle pyruvate vs. muscle oxygen content

• Minute volume vs. muscle glucose

• Lung compliance vs. PEEP

• Lung compliance vs. muscle pyruvate

We presented potential physiological interpretations for these results as well. All but one of these variable pairs directly involve parameters for treatment/ventilation chosen by physicians in the ICU. Therefore, we can also conclude that the effects of treatment on physiological measurements changes when infections are contracted in the intensive care unit. Some of these differences have already been reported in the literature while others are novel. This shows that our technique can be used to discover previously unknown relationships between physiologic variables. This work points to opportunities to study the changes that have not yet been reported, potentially opening new doors to discover how our best efforts to heal patients can both alter and be informed by their physiology.

## Competing interests

The authors declare that they have no competing interests.

## Authors' contributions

AG and AB conceived of this study. AG performed the analysis and drafted the manuscript. MC and GM participated in data collection and interpretation of results, and conceived of the original study to generate these data. AB participated in the analysis and interpretation of the results. All authors edited and approved the final manuscript.
